# Excellent electrical conductivity of the exfoliated and fluorinated hexagonal boron nitride nanosheets

**DOI:** 10.1186/1556-276X-8-49

**Published:** 2013-01-24

**Authors:** Yafang Xue, Qian Liu, Guanjie He, Kaibing Xu, Lin Jiang, Xianghua Hu, Junqing Hu

**Affiliations:** 1 State Key Laboratory for Modification of Chemical Fibers and Polymer Materials, College of Materials Science and Engineering, Donghua University, Shanghai, 201620, China

**Keywords:** Boron nitride, Exfoliation, Fluorination, Electrical conductivity

## Abstract

The insulator characteristic of hexagonal boron nitride limits its applications in microelectronics. In this paper, the fluorinated hexagonal boron nitride nanosheets were prepared by doping fluorine into the boron nitride nanosheets exfoliated from the bulk boron nitride in isopropanol via a facile chemical solution method with fluoboric acid; interestingly, these boron nitride nanosheets demonstrate a typical semiconductor characteristic which were studied on a new scanning tunneling microscope-transmission electron microscope holder. Since this property changes from an insulator to a semiconductor of the boron nitride, these nanosheets will be able to extend their applications in designing and fabricating electronic nanodevices.

## Background

Innovative and constructive doping into nanomaterials has attracted considerable attention, because a specific dopant could bring a revolutionary change on the materials’ properties and applications, such as in the fields of energy storage [1,2], photovoltaics [3,4], and biosensor [5]. Graphene exfoliated from graphite is a good example, which is doped by some elements (e.g., N [6,7] and B [6,8]) has been explored many fascinating properties and applications. The hexagonal boron nitride nanosheets (h-BNNSs) are a structural analogue of graphene, so-called ‘white-graphene’ [9], in which B and N atoms alternatively substitute for C atoms [10]. However, in contrast to the comprehensive researches on graphene [6,11-13], especially the breakthrough in semiconductor devices [14,15], the study on h-BNNSs, including their exfoliation, properties (by doping or functionalizing), and applications, is in its infancy. This may attribute to the ‘lip-lip’ ionic characteristic of the bonding between neighboring boron nitride (BN) layers [10], which is stronger than the weak Van der Waals force between graphene layers and the wide band gap of h-BNNS (approximately 4–6 eV) [16], making it as an insulator. If the two aforesaid challenging problems are solved, h-BNNS will exhibit more novel properties and applications in nanoelectronics and nanophotonics. Of particular interest is that minishing the band gap of h-BNNS by doping into some featured elements could lead an amazing change from an insulator to a semiconductor.

Doping preferentially takes place at the more vulnerable sites, so it will be much easier to perform doping experiment with fewer-layered h-BNNSs. Though several methods have been presented to prepare few-layered or mono-layered h-BNNSs [17,18], the rigorous conditions restrict these methods to be widely conducted. Recently, Golberg [19] and Coleman et al. [20] have put forward a facile route to few-layered or mono-layered h-BNNSs by sonicating the bulk BN in a common liquid solvent. Speaking of doping, several methods have been reported such as placing peculiar dopant into well-defined regions of h-BN nanotubes (h-BNNTs). Wei et al. [21] used the electron-beam-induced strategy and Wang et al. [22] applied the noncovalent functionalization method to dope carbon (C) into the h-BNNTs, which demonstrated the electrical conductivity increased with the C content. In comparison with C, doping of fluorine (F) may be a new pathway to regulate the electrical properties of h-BN. Since F is a highly electronegative element and has excessive valence electrons compared to B and N, doping F into some nanomaterials should reliably yield a p-type semiconductor at low coverages and even a conductor at high coverages [23,24]. Some theoretical calculations have predicted the possible functions of doping F into h-BNNTs and h-BNNSs [24-26]. Only Tang et al. [23] reported the electrical conductivity of h-BNNTs which were fluorine-functionalized during the nanotubes’ growth. Doping F into h-BNNSs and examining their corresponding electrical properties have not been realized experimentally. Therefore, it is of crucial importance to develop a facile method for doping F into h-BNNSs and explore its electrical properties.

Herein, we doped F into few- and mono-layered h-BNNSs and first pursued their electrical properties with the scanning tunneling microscope-transmission electron microscope (STM-TEM) holder. The few-layered h-BNNSs were exfoliated from the bulk BN using a modified chemical solution route in isopropanol (IPA) at 50°C and with ultrasonicating, and subsequently fluorinated with a solution of fluoboric acid (HBF_4_). The fluorinated h-BNNSs exhibit a significant characteristic of a semiconductor, with a current up to 15.854 μA.

## Methods

All chemicals were purchased from Sinopharm Chemical Reagent Co. Ltd. (Shanghai, China) and used without further purification.

### Exfoliation of bulk BN to few-layered or mono-layered h-BNNSs

In a typical exfoliation process, the bulk boron nitride (BN) powders (0.25 g) were dispersed in a solvent of IPA contained in a 100-mL round-bottomed flask, and then as-formed solution was heated at 50°C for 24 h under magnetic stirring. Subsequently, the solution was subjected to further ultrasonication for 20 h in a low power sonic bath. Then the resulted solution in the flask was stood for 2 days, and the supernatant solution was removed to the centrifugal tube followed by centrifugation at 14,000 rpm for 10 min. Afterwards, the precipitate was washed with acetone several times to remove the IPA absolutely and dried at 60°C overnight. Finally, a milk-white solution of few-layered and mono-layered h-BN nanosheets (h-BNNSs) were obtained.

### Fluorination of h-BNNSs

In a representative fluorination experiment, as-prepared h-BN nanosheets (0.25 g) and HBF_4_ (50 mL) were mixed in a 100-mL round-bottomed flask. Then the mixture was heated at 50°C for 8 h under magnetic stirring. After this treatment, the mixture was cooled to room temperature naturally. Finally, the fluorinated products were removed to the centrifugal tube, washed with deionized water several times, and dried at 60°C for several hours.

#### Characterizations

The morphologies and structures of the exfoliated and fluorinated products were characterized by a field-emission scanning electron microscope (FE-SEM, Hitachi S-4800, Tokyo, Japan) equipped with an X-ray energy-dispersive spectrometer (EDS), a transmission electron microscope (TEM, JEOL, JEM-2010F, JEOL Ltd., Tokyo, Japan), an atomic force microscope (AFM, NanoScope IV Veeco Instruments Inc., Plainview, NY, USA), and a D/max-2550 PC powder X-ray diffractometer (XRD, Rigaku Co., Tokyo, Japan). X-ray photoelectron spectroscopy (XPS) spectra were conducted on an Axis Ultra DLD X-ray photoelectron spectroscopy (Kratos Co., Manchester, UK). Fourier transform infrared (FTIR) spectroscopy investigations were performed on an IR Rrestige-21 FTIR spectrometer (Shimadzu Co., Kyoto, Japan).

## Results and discussion

**Figure 1:**
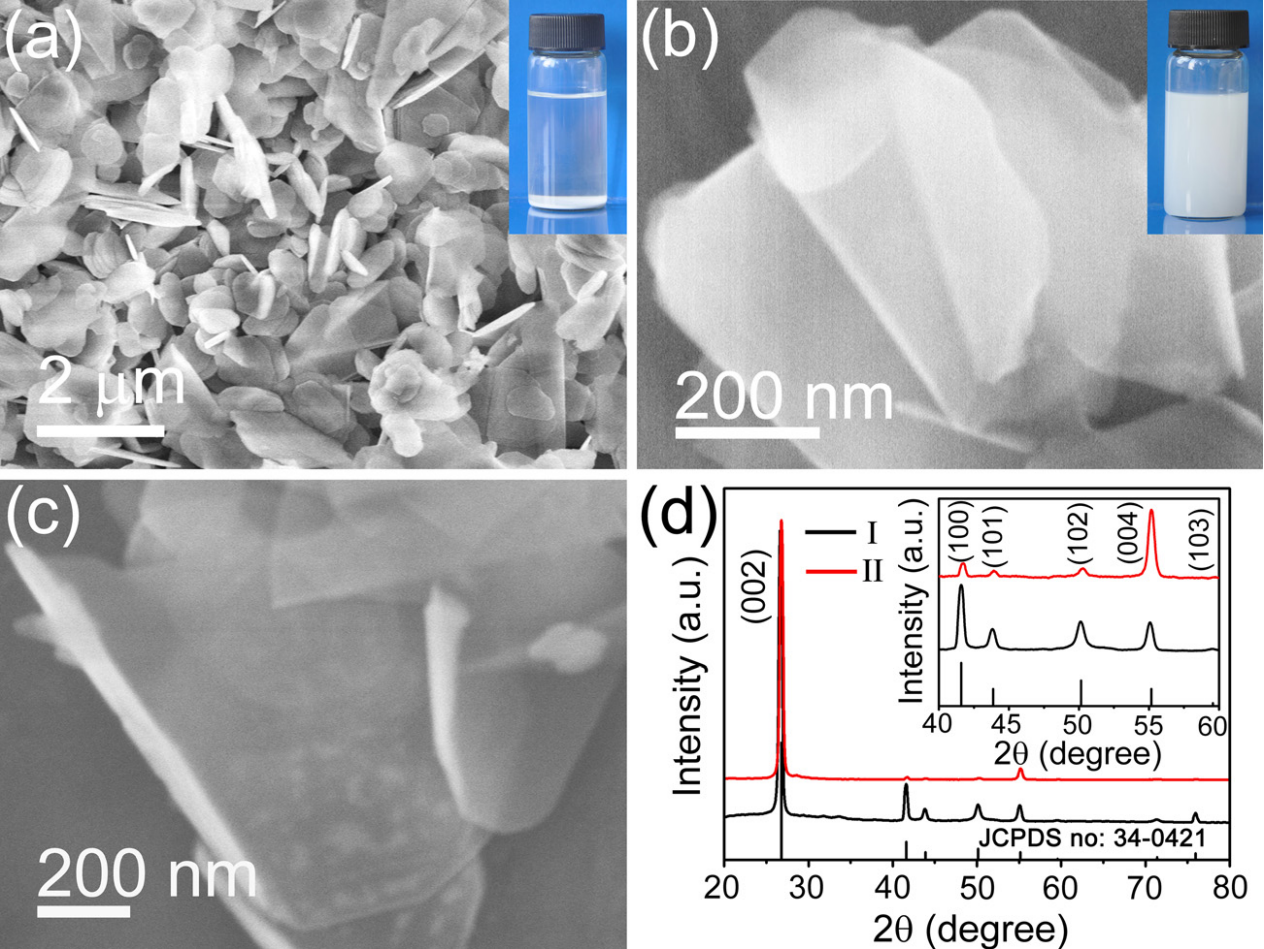
**Overall morphological characterization and XRD analysis of the precursor and exfoliated products.** (**a**) SEM image of the precursor bulk BN, an inset of a photograph showing the precursor dispersed in IPA. (**b, c**) SEM images of exfoliated products, an inset in b of a photograph showing the exfoliated products dispersed in IPA standing for two weeks. (**d**) XRD patterns of the bulk BN (I) and exfoliated products (II), respectively, referring to the JCPDS file of the standard BN powders, an inset showing the amplified patterns.

Comparatively, three solvents (IPA, dimethyl sulfoxide (DMSO), and *N*-methyl pyrrolidone (NMP)) were used to exfoliate the bulk BN for producing BNNSs. The detailed characterization and analysis are given in Figure S1 in Additional file 1. It is found that under our experimental conditions, the IPA is a better polar solvent to peel off the bulk BN among them. Figure 1 shows the low- and high-magnification FE-SEM images and XRD patterns of the bulk BN powders and exfoliated products using the IPA as the solvent. The low-magnification SEM image in Figure 1a presents the overall morphology of the precursor, which demonstrates that the bulk BN powders consist of irregular shapes and a few of thick flakes with lateral sizes ranging from hundreds of nanometers to several micrometers. The high-magnification SEM images in Figure 1b,c reveal the sufficient exfoliation of the bulk BN. Clearly, both the thickness and lateral sizes of the exfoliated products are decreased, forming h-BNNSs. Figure 1b shows the few-layered h-BNNSs which appear like the booming flowers and Figure 1c demonstrates the BN nanosheets with a rolling up edge. In addition, the two upper insets of photographs in Figure 1a,b show the precursor (a) and exfoliated products (b) both dispersed in IPA. It is found that the milk-white solution of the h-BNNSs can remain stable for a long period, even more than 2 weeks. This is mainly because the exfoliated products are too thin to deposit, suggesting the sufficient peeling of the bulk BN by the presented chemical method. Comparatively, the precursor BN powders in the solution completely deposited on the bottom of the bottle in several minutes, leaving a transparent solution, which is clearly due to the large lateral sizes of the bulk BN precursor. In the XRD sample preparation process, in order to make the preferential orientation (002) planes on the holder as much as possible, the XRD sample was prepared as follows. First, the white powders of as-prepared BN nanosheets were dissolved in the ethanol with ultrasonic dispersion. Second, the dispersing solution was dropwise added on a glass holder which was cleaned by ethanol. Lastly, the glass coated by the dispersing solution was dried at 60°C to evaporate the ethanol and form a thin film of these BN nanosheets for the XRD characterization. Shown (including its inset) in Figure 1d is comparative XRD patterns of the bulk BN powders (I), exfoliated products (II), respectively, referring to the Joint Committee on Powder Diffraction Standards (JCPDS card number 34–0421) (bottom) for the standard h-BN powders. All of the diffraction peaks from the products can be readily indexed to the h-BN with lattice constants of *a* = *b* = 2.504 and *c* = 6.656 Å. A series of intensive peaks are at 2θ = 26.764°, 41.597°, and 55.164°, with *d*-spacing of 3.328, 2.169, and 1.663 Å, corresponding to the (002), (100), and (004) planes of the h-BN, respectively, in which (004) plane is parallel to (002) plane. From the amplified patterns in its inset, the intensity of the (004) plane from the exfoliated products is unusually intensive, by analyzing the intensity (*I*) ratio between (100) and (004) planes. It could visually indicate a very efficient exfoliation from the bulk BN powders by the present route. In black curve I, the *I*_100_/*I*_004_ is approximately 2; however, in red curve II, the *I*_100_/*I*_004_ is only approximately 0.25 (or the *I*_004_/*I*_100_ reaches up to approximately 4). As the h-BNNSs have a tendency to lie on their widest facets when they were dispersed randomly in a glass sample holder, the widest facets were the preferential orientations, i.e., the (002) (or 004) planes in the XRD measurement. In fact, the exposed (002) crystal surface of a h-BN crystal likes the (002) plane of graphite [27], the exfoliation process will occur on the (002) plane, which would be valuable to exploit more excellent properties of h-BNNSs.

**Figure 2:**
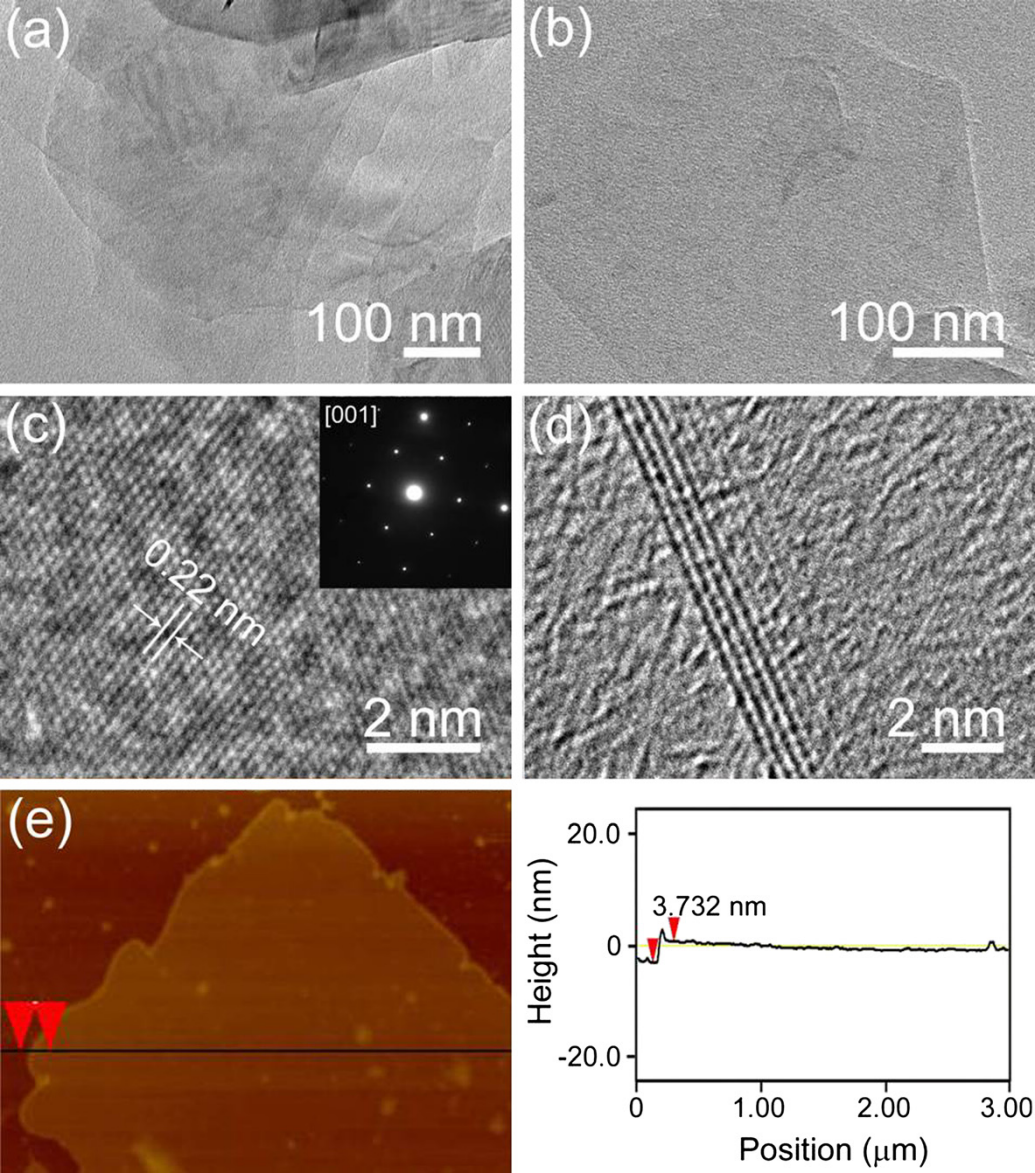
**TEM and AFM imaging characteristics of the exfoliated products.** (**a,b**) TEM images of as-exfoliated few-layered and mono-layered h-BNNSs, respectively. (**c**) HRTEM image of the BNNS, an inset showing its corresponding SAED pattern along the [001] axis. (**d**) HRTEM image displaying this BN nanosheet with a thickness of around 2.67 nm. (**e**) AFM image and the corresponding height profile of a BNNS.

Transmission electron microscopy (TEM) (Figure 2a,b,c,d) and AFM (Figure 2e) images further present the characteristics of the exfoliated products. Figure 2a shows few-layered h-BNNSs covering the carbon film, in which the top layers are transparent to the electron beam to see the bottom layers. Figure 2b gives an image of mono-layered h-BNNS. The high-resolution TEM (HRTEM) image in Figure 2c demonstrates the hexagonal lattice structure of the h-BNNSs, in which the marked white line clearly shows the measured *d* spacing of 0.22 nm, nearly equaling to the distance of the (100) planes. As suggested by the selected area electron diffraction (SAED) pattern (Figure 2c, inset), which was taken with electron beam along [001] zone axis, perpendicular to the surface of this nanosheet, it reveals the well-crystallized nature and the hexagonal structure characteristic of the exfoliated products. The layers of h-BNNSs can be directly calculated by examining the folded edges with HRTEM imaging. As illustrated in Figure 2d, it provides a typical multi-layered h-BNNSs with a width of around 2.67 nm (approximately eight BN (002) layers), corresponding to a distance of the adjacent layers of 0.33 nm, which is quite close to the *d*_002_ (0.3328 nm) of BN material. The nanosheet edge is clean and abrupt on an atomic scale, and there is no amorphous layer covering on its surface. Furthermore, we applied AFM and the corresponding height profile to examine the surface nature and to estimate the thickness of the h-BNNSs (Figure 2e). It is found that the surface of this sheet is rather flat and its height is 3.732 nm (approximately 11 BN (002) layers). The more detailed AFM measurements are given in Figure S4 in Additional file 1.

**Figure 3:**
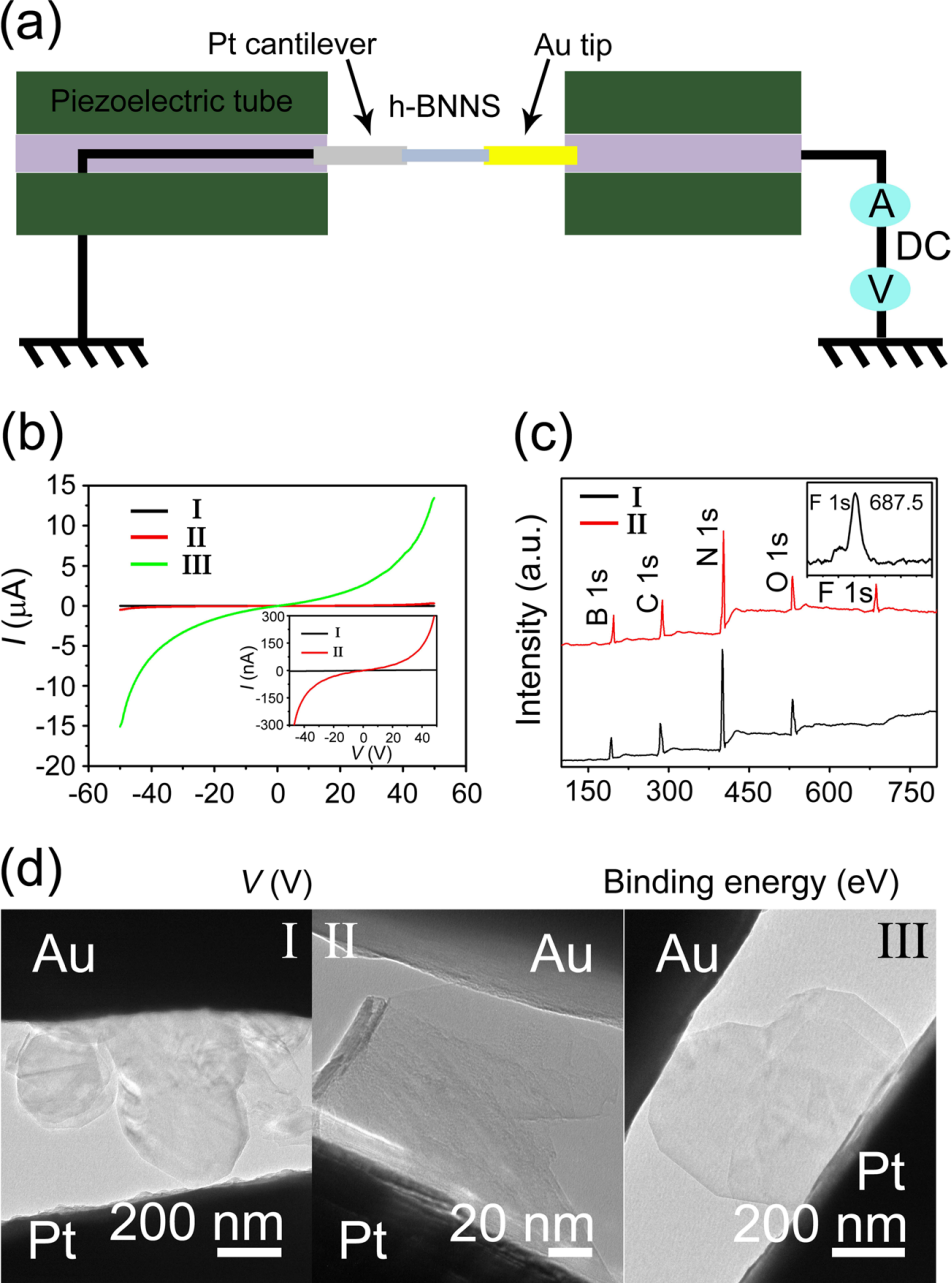
**Schema of electrical measurement, *I*-*V *characteristic curves,** X**PS spectra, and TEM images.** (**a**) Schematic illustration of the electrical measurement setup based on the STM-TEM holder. (**b**) Current–voltage (*I*-*V*) characteristic curves of bulk BN (I), the exfoliated (II), and fluorinated (III) BNNS, respectively; an inset showing the amplified view of the *I*-*V* curves (I and II). (**c**) XPS spectra of the exfoliated (I) and fluorinated (II) BNNS, respectively, an inset showing F 1s region. (**d**) TEM images of bulk BN (I), the exfoliated (II) and fluorinated (III) BNNS connected between the Pt cantilever and Au tip, respectively.

After fluorination of the h-BN nanosheets, we studied their electrical conductivities performed on a new STM-TEM holder commercialized by Nanofactory Instruments AB (Gothenburg, Sweden), which was arranged within a 200-kV field emission high-resolution TEM (JEM-2010F), which has been described in elsewhere [28]. The schematic of the experimental setup is represented in Figure 3a, as described in our previous studies [29]. Briefly, an Au tip is attached to a fixed electrical sensor, and a Pt cantilever adhering with a little of the fluorinated products is placed on the piezo-movable side of the holder. Firstly, the relative position of Au tip and Pt cantilever is manually adjusted with tweezers under an optical microscope to get a minimal possible gap between them, which can be distinguished by eyes. Then the location of Au tip and a fluorinated BN nanosheet is modulated through the nanoscale precision piezo-driven manipulator of STM-TEM holder to build a BN bridge circuit (Figure 3d, III). Finally, a PC-compatible software automatically coordinates the final stages and controls the nanosheets displacement and movement rate. On the basis of the model adopted from the classical electricity, the electrical conductivity of this fluorinated BNNS (III) was measured by the dedicated software and electronics from Nanofactory Instruments AB. To make a careful comparison, the electrical conductivities of the precursor bulk BN (I) and the original exfoliated products (II) were also measured. The TEM images of bulk BN and the exfoliated BNNS connected between the Pt cantilever and Au tip are given in Figure 3d (I) and (II), respectively. The typical *IV* characteristic curves are shown in Figure 3b, in which an applied voltage arranges from −50 to 50 V. As we expect, the fluorinated BN nanosheets display a typical semiconductor characteristic of the *IV* curve (green), and its current value varies from −15.854 to 13.663 μA. While the precursor bulk BN shows its intrinsic electric insulation characteristic with no detectable current under the same bias voltage (black). The current value of the h-BNNSs without fluorination ranges from −300 to 300 nA (red, as shown by a magnified inset), which may owe to the indirect to direct bandgap transition [30]. The fluorinated h-BNNSs possessing an excellent electrical conductivity suggest that the BN material is transformed from the insulator to a semiconductor through the effective doping of F, which will extend their applications in nanoelectronics.

In order to further identify doping F into the h-BNNSs, we analyzed the chemical composition of the products by XPS (Figure 3c) and EDS (Figure S5 in Additional file 1). Figure 3c shows the XPS spectra of the exfoliated (I) and further fluorinated (II) products, respectively. The results reveal that B, C, N, O and F elements exist in the fluorinated products, in which the binding energy of B 1s, C 1s, N 1s, O 1s, and F 1s is corresponding to 197.6, 288.4, 401.7, 530.0, and 686.6 eV, respectively. The existence of C and O elements commonly seen could attribute to the carbon contamination and water adsorbing from the atmosphere. Comparatively, the curve I only show an existence of the B, C, N and O elements. It suggests the F element appearing in the fluorinated products is the key factor contributing to the excellent electrical conductivity of the h-BNNSs. If the F only attaches to the surface of BNNSs, it will be too unstable to exist under the beam irradiation in the electron microscope [23,24], resulting in electrical conductivity that will not be significantly improved. So, we deduce that the excellent electrical conductivity of the fluorinated BN nanosheets alternatively confirms the F was doped into the few-layered h-BNNSs successfully.

## Conclusions

In summary, an excellent electrical conductivity of the exfoliated and fluorinated h-BNNSs, i.e., transferring from the insulator to the semiconductor, has been reported. A facile chemical route was developed to exfoliate the bulk BN into few- and mono-layered h-BNNSs, then a simple chemical solution route successfully fluorinated the BNNSs. Importantly, the fluorinated BNNSs possesses the excellent electrical property with a current up to 15.854 μA, showing a typical semiconductor characteristic, which will open a new opportunity in designing and fabricating electronic nanodevices.

## Competing interests

The authors declare that they have no competing interests.

## Authors’ contributions

YX carried out the exfoliation and fluorination and drafted the manuscript. QL, GH, KX, LJ, and XH participated in discussion of the study. YX and JH participated in the design of the study and performed the statistical analysis. YX and JH conceived of the study, and participated in its design and coordination. All authors read and approved the final manuscript.

## Supplementary Material

Additional file 1: Supporting information: figures showing further XRD, FTIR, AFM and EDS data. Click here for file
