# Supplementary material for: Excellent electrical conductivity of the exfoliated and fluorinated hexagonal boron nitride nanosheets
Source: Nanoscale Res Lett. 2013 Jan 24;8(1):49. doi: 10.1186/1556-276X-8-49 (PMC3564689; doi:10.1186/1556-276X-8-49)
Supplement: Additional file 1: — Supporting information: figures showing further XRD, FTIR, AFM and EDS data. [file 1556-276X-8-49-S1.doc]

**Supporting Information**

**Excellent electrical conductivity of the exfoliated and fluorinated hexagonal boron nitride nanosheets**

Yafang Xue, Qian Liu, Guanjie He, Kaibing Xu, Lin Jiang, Xianghua Hu and Junqing Hu*

State Key Laboratory for Modification of Chemical Fibers and Polymer Materials, College of Materials Science and Engineering, Donghua University, Shanghai, 201620, China

Author e-mail address: [hu.junqing@dhu.edu.cn](mailto:hu.junqing@dhu.edu.cn)

**Figures**


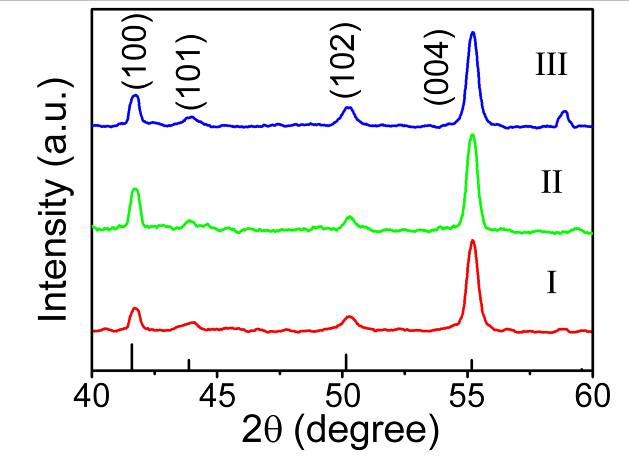


Figure S1 Compared XRD patterns of the products exfoliated by a solvent of IPA (I), DMSO (II) and NMP (III), respectively.

In our experiment, the few-layered and mono-layered h-BNNSs were exfoliated from the bulk BN in a solvent of IPA, DMSO (dimethyl sulfoxide) and NMP (N-methyl pyrrolidone), respectively, via a modified chemical solution route. The exposed (002) crystal surface of the h-BN crystal likes the (002) plane of graphite, and the exfoliation process will occur on the (002) plane[1], in which the appropriate surface tension of the solvent minimizes the energy of exfoliation[2]. Here, we analyzed the intensity ratio between (100) and (004) planes from different products exfoliated by the IPA (I), DMSO (II) and NMP (III) under the same synthetic and XRD measurement conditions. The *I*_004_ / *I*_100_ was found to be 3.13, 1.96 and 2.56 of them, respectively, referring to the JCPDS file (card no.: 34-0421) of the standard BN powders. It suggests that as-exfoliated products using the solvent of IPA have a more significantly preferential orientation along (002) or (004) plane, as compared with the products by using the other two solvents, that is, the IPA is the best polar solvent peeling off the bulk BN among the three solvents in our experimental conditions.


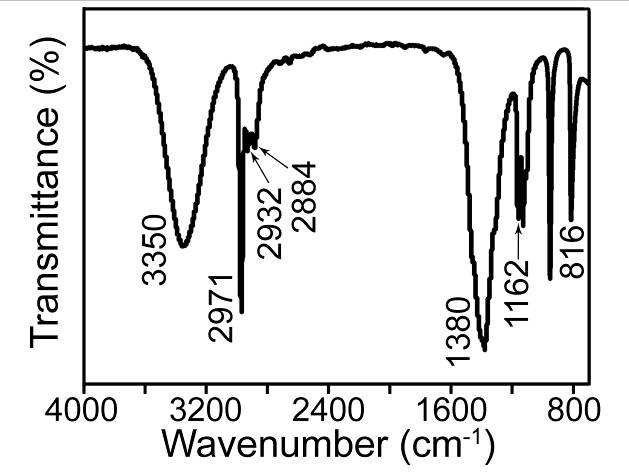


Figure S2 FTIR spectrum of the mixture system of BN nanosheets and IPA.

A mixture of the BN nanosheets and IPA obtained from the above chemical solution route was investigated by the FTIR spectrum to examine the interaction between them. The peaks at 1381 and 817 cm-1 are assigned to the stretching (ν) and bending (δ) B–N modes of BN [1,3], while other peaks coming from O-H (3350 cm-1), C-H (2971, 2932 and 2884 cm-1) and C-O (1162 cm-1) groups can also be observed, which can directly suggest that the h-BNNSs are successfully functionalized by the IPA.


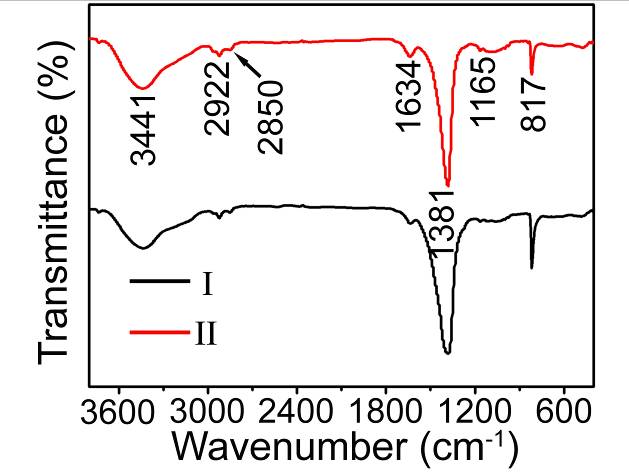


Figure S3 FTIR spectra of the precursor bulk BN (I) and exfoliated products (II).

In two FTIR curves, except the peak of adsorbing water at 3441 cm-1, only two peaks are observed at 1381 and 817 cm-1, attributing to the ν and δ B–N modes of h-BN. It suggests that the organic solvent of IPA used during the exfoliation process was washed away completely and also further confirms that the as-prepared products are free of the foreign groups.


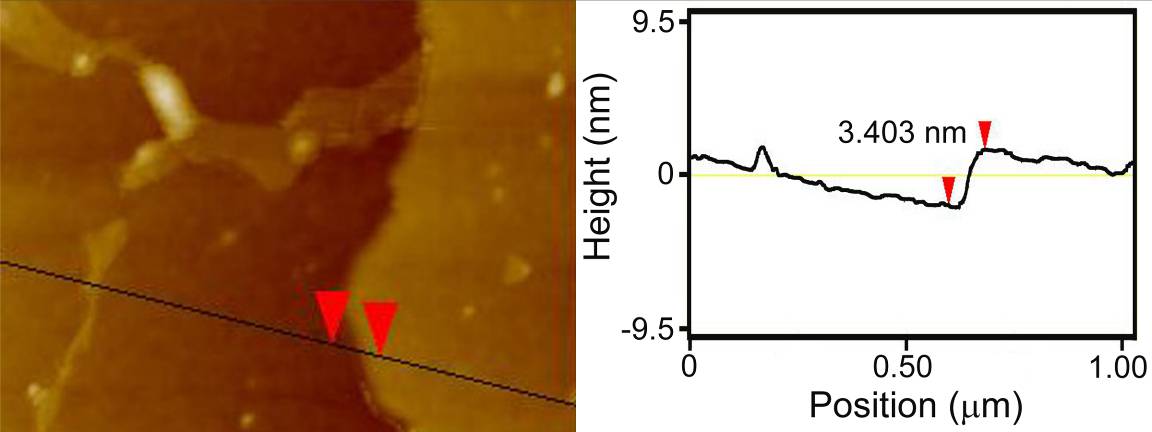


Figure S4 AFM image and the corresponding height profile of a BNNS.

The typical height value of the h-BNNS is estimated to be ~ 3.403 nm, corresponding to ~ 10 BN (002) layers.


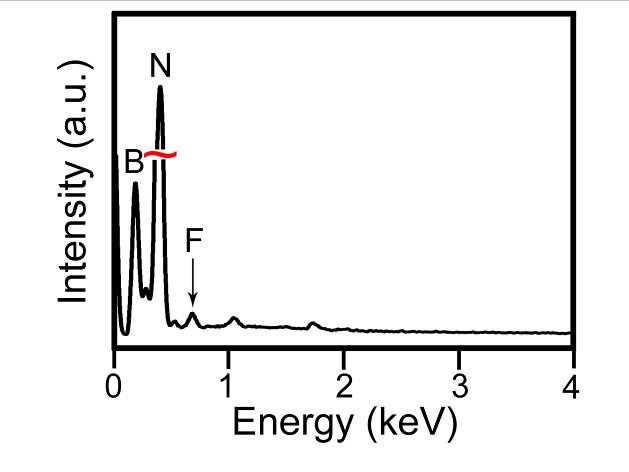


Figure S5 EDS spectrum of the fluorinated products.

Though the elements of B, N and F can be detected by the EDS system, each quantity for them (particular these light elements) can not be exactly given [4].


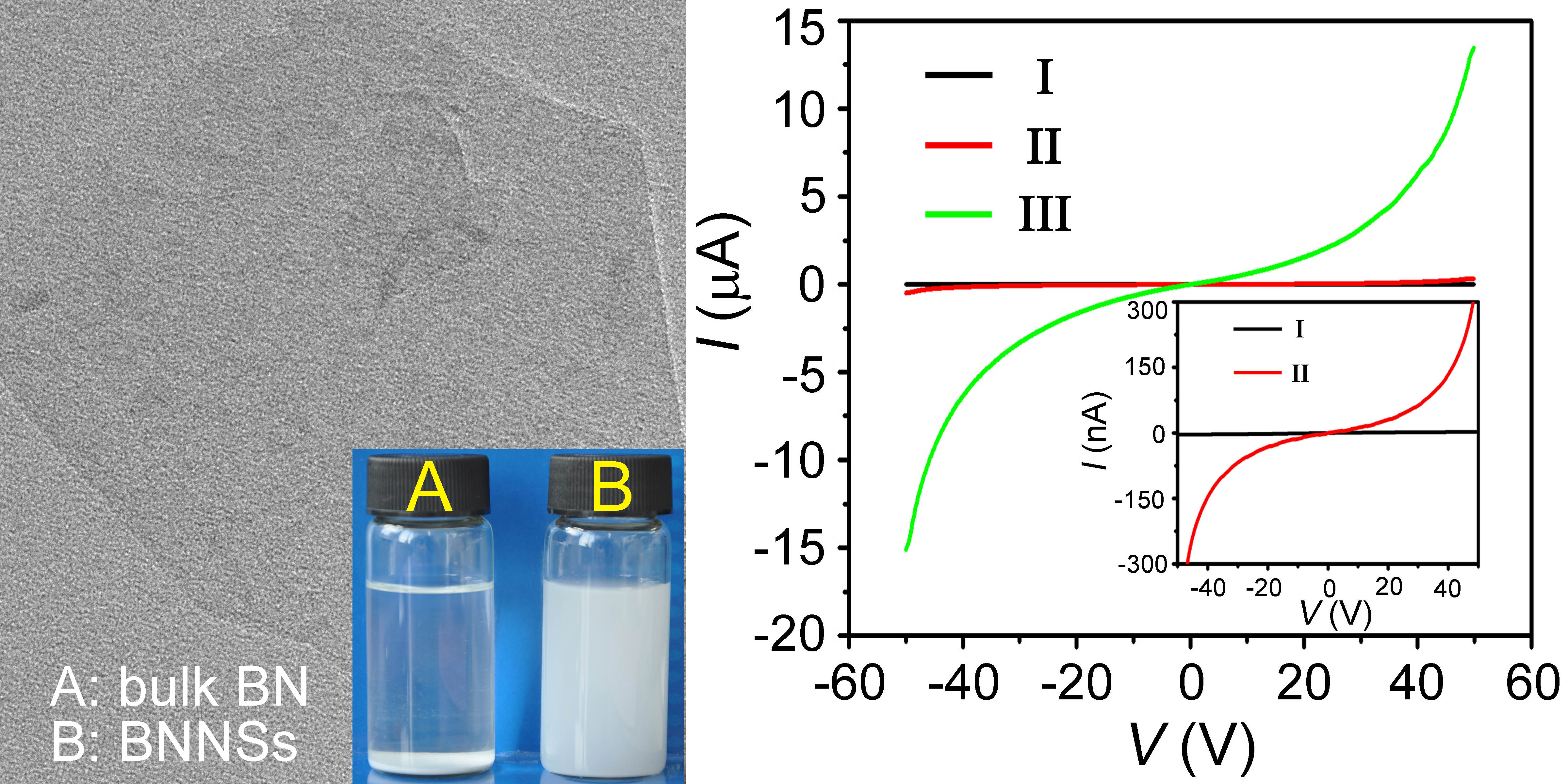


The fluorinated h-BN nanosheets, which were obtained by doping F into the h-BNNSs exfoliated from the bulk BN via a facile chemical solution route, possess an excellent electrical conductivity showing a typical semiconductor characteristic, which will extend their applications in nanoelectronics.

**References**

1. Zhi CY, Bando Y, Tang CC, Kuwahara H, Golberg D: **Large‐Scale Fabrication of Boron Nitride**

**Nanosheets and Their Utilization in Polymeric Composites with Improved Thermal and Mechanical Properties.** *Adv. Mater* 2009, **21**:2889.

2. Coleman JN, Lotya M, O’Neill A, Bergin SD, King PJ, Khan U, Young K, Gaucher A, De S, Smith RJ, Shvets IV, Arora SK, Stanton G, Kim HY, Lee K, Kim GT, Duesberg GS, Hallam T, Boland JJ, Wang JJ, Donegan JF, Grunlan JC, Moriarty G, Shmeliov A, Nicholls RJ, Perkins JM, Grieveson EM, Theuwissen K, McComb DW, Nellist PD, Nicolosi V: **Two-dimensional nanosheets produced by liquid exfoliation of layered materials.** *Science* 2011, **331**:568.

3. Lian G, Zhang X, Tan M, Zhang SJ, Cui DL, Wang QL: **Facile synthesis of 3D boron nitride nanoflowers composed of vertically aligned nanoflakes and fabrication of graphene-like BN by exfoliation.** *J. Mater. Chem* 2011, **21**:9201.

4. Tang CC, Bando Y, Huang Y, Yue SL, Gu CZ, Xu FF, Golberg D: **Fluorination and electrical conductivity of BN nanotubes.** *J. Am. Chem. Soc* 2005, **127**:6552.
